# Complications After Bariatric Surgery: Insights from a 14-Year Single-Institutional Study Without Fistula

**DOI:** 10.3390/jcm15010095

**Published:** 2025-12-23

**Authors:** Mădălina Maxim, Petru Radu Soroceanu, Vlad Ionuț Vlasceanu, Bogdan Galuscă, Raoul Vasile Lupușoru, Alin Constantip Pînzariu, Alina Onofriescu, Lucian Ambrosie, Gheorghe Balan, Mihaela Toader, Irina Mihhaela Abdulan, Bogdan-Mihnea Ciuntu, Daniel Vasile Timofte

**Affiliations:** 1 Department of General Surgery, Faculty of Medicine, “Grigore T. Popa” University of Medicine and Pharmacy, 16 Universitatii Street, 700115 Iasi, Romania; madalynamaxim@yahoo.com (M.M.); petru.soroceanu@umfiasi.ro (P.R.S.); vlasceanu.vlad@yahoo.com (V.I.V.); lucian.ambrosie@hse.ie (L.A.); daniel.timofte@umfiasi.ro (D.V.T.); 2 General Surgery Clinic, “St. Spiridon” County Emergency Clinical Hospital, 1 Independence Boulevard, 700111 Iasi, Romania; 3 Department of Endocrinology, Diabetes, Metabolism and Eating Disorders, Centre Hospitalier Universitaire de Saint-Etienne, Cedex 2, 42055 Saint-Etienne, France; bogdan.galusca@chu-st-etienne.fr; 4 Department of Pathophysiology, Morphofunctional Sciences II, Faculty of Medicine, “Grigore T. Popa” University of Medicine and Pharmacy, 700115 Iasi, Romaniaalin.pinzariu@umfiasi.ro (A.C.P.); 5 Unit of Diabetes, Nutrition, and Metabolic Diseases, Faculty of Medicine, “Grigore T. Popa” University of Medicine and Pharmacy, 700115 Iasi, Romania; 6 Department of Gastroenterology Clinical Emergency Hospital St. Spiridon, “Grigore T. Popa” University of Medicine and Pharmacy, 700111 Iasi, Romania; gheorghe-g-balan@umfiasi.ro; 7 Department of Medical Specialties I, “Grigore T. Popa” University of Medicine and Pharmacy, 700115 Iasi, Romania

**Keywords:** obesity, bariatric surgery, bariatric surgery complications, without fistula

## Abstract

**Background/Objectives**: Obesity imposes a significant and growing burden on healthcare systems worldwide. Bariatric surgery remains the most effective long-term treatment for morbid obesity, but its success depends heavily on the quality of perioperative management and institutional expertise. This study presents a comprehensive analysis of 14 years of bariatric surgical activity in a university-based Center of Excellence, emphasizing complication rates and safety outcomes. **Methods**: A cohort analysis was performed on a prospectively collected database including all bariatric procedures conducted between June 2012 and June 2025 in an Obesity Center, located in ‘Saint Spiridon’ Hospital’ in Iasi, Romania. Eligibility was determined according to the American Society for Metabolic and Bariatric Surgery (ASMBS) and International Federation for the Surgery of Obesity and Metabolic Disorders (IFSO) guidelines. All patients underwent standardized preoperative evaluation by a multidisciplinary team. **Results**: Over a 14-year period, 1010 patients underwent surgery and had a mean age of 39 years and 72% of them were females. A total of 68 patients (6.73%) experienced complications, including 28 (2.77%) within 30 days and 40 (3.96%) after first month. No postoperative fistulas or deaths were recorded during the entire study period. **Conclusions**: The long-term data from over one thousand consecutive bariatric cases confirm the high safety and effectiveness of a multidisciplinary, protocol-driven approach. The absence of postoperative fistulas and mortality underscores the value of institutional experience and standardized perioperative care.

## 1. Introduction

Obesity, characterized by pathological and sustained weight gain, is currently recognized as a chronic, relapsing, and multifactorial disease with substantial metabolic and psychosocial implications. It represents a major etiological factor in the global increase of several diseases and beyond its physical impact, individuals affected by overweight and obesity frequently experience significant psychosocial burden, encompassing depression, impaired self-esteem, and social discrimination with a great impact on quality of life. The combined physical, psychological, and societal consequences of obesity also generate additional indirect economic losses through reduced work productivity, increased disability rates, and premature mortality [1,2].

According to the World Health Organization (WHO), in 2022, approximately one in eight individuals were affected by obesity. Since 1990, the prevalence of obesity has quadrupled among adolescents and doubled among adults. An estimated 2.5 billion adults (≥18 years) were classified as overweight in 2022, of whom 43% had a body mass index (BMI) within the overweight range and 16% met the diagnostic criteria for obesity. Additionally, 37 million children under the age of 5 and over 390 million individuals aged 5–19 were reported as overweight or obese [3].

Obesity is defined as a chronic, multifactorial disease characterized by abnormal or excessive fat accumulation, posing serious health risks. It is strongly associated with metabolic disorders (such as type 2 diabetes), cardiovascular diseases, certain cancers, and impaired bone, reproductive, and psychological health, ultimately diminishing overall quality of life [4].

**Diagnosis and Classification of Overweight and Obesity**

The diagnosis of overweight and obesity is based on the assessment of body mass index (BMI), calculated using the formula: weight (kg)/height^2^ (m^2^). BMI represents a standardized anthropometric index used to estimate excess adiposity, while complementary measurements—such as waist circumference or waist-to-hip ratio—may provide additional information regarding central fat distribution and associated metabolic risk. Classification vary by age and sex in pediatric populations. In adults, the World Health Organization (WHO) defines overweight as a BMI ≥ 25 kg/m^2^ and obesity as a BMI ≥ 30 kg/m^2^ [3].

**Epidemiological Trends**

In 2022, an estimated 2.5 billion adults aged ≥ 18 years were classified as overweight, including over 890 million individuals with obesity. This prevalence corresponds to approximately 43% of the global adult population, with overweight affecting 43% of men and 44% of women. These figures represent a substantial increase [3]. The prevalence of excess body weight varied by region, ranging from 31% in the South-East Asia and African Regions to 67% in the Region of the Americas [5,6,7]. Overweight and obesity are rising rapidly across the European Union, with 52.7% of adults (≥18 years) classified as overweight in 2019. Prevalence was similar between sexes and increased with age, ranging from 25.0% in young adults (18–24 years) to 65.7% in older adults (65–74 years) [8,9].

In Romania, only about 40% of individuals aged ≥10 years have a normal body weight, while the rest are at least overweight, with approximately 25% meeting criteria for obesity. Higher obesity prevalence is observed among adults over 45 years, married individuals, those from low- to middle-income households, and lower educational status, with class I obesity predominating in men. In children aged 10–15 years, overweight and obesity are less common than in adults, affecting 16% in each category [10,11].

**Causes of Overweight and Obesity**

Overweight and obesity are the consequences of a persistent energy imbalance, characterized by caloric intake surpassing energy expenditure. Although these conditions are typically multifactorial, influenced by environment, psychosocial factors, and genetic predispositions, certain instances may exhibit identifiable etiologies. These can include pharmacological agents, underlying medical conditions, prolonged physical inactivity, iatrogenic factors, or genetic syndromes [12]. Elevated BMI was linked to nearly 5 million deaths globally in 2019 due to noncommunicable diseases (NCDs), including cardiovascular diseases, diabetes, cancers, neurological disorders, chronic respiratory diseases, and digestive system conditions [13]. Childhood and adolescent obesity is associated with an earlier onset and increased risk of non-communicable diseases (NCDs) like type 2 diabetes mellitus and cardiovascular disorders. It also results in substantial psychosocial impacts, including reduced academic achievement, social stigma, discrimination, and bullying, with a high probability of continuation into adulthood [14,15,16,17,18].

**Bariatric Surgery Overview**

The global economic burden of obesity is substantial and projected to rise without effective public health interventions. According to the International Federation for the Surgery of Obesity and Metabolic Disorders (IFSO), over 480,000 metabolic and bariatric procedures were reported across 24 national and 2 regional registries, representing 81.4% of contributing datasets. Bariatric surgery has evolved into a standardized, low-risk intervention, with most national registries reporting perioperative mortality rates below 1%, underscoring its safety and efficacy in appropriately selected patients [19,20].

Sleeve gastrectomy (SG) is currently the most frequently performed bariatric procedure worldwide, followed by Roux-en-Y gastric bypass (RYGB) and one-anastomosis gastric bypass (OAGB). Additional bariatric interventions include single-anastomosis duodeno-ileal bypass with sleeve gastrectomy (SADI-S), biliopancreatic diversion (BPD), adjustable gastric banding (AGB), and various endoscopic techniques [21,22,23]. The proportion of “other” procedures, especially in revisional surgery, is growing steadily. Most bariatric surgeries are performed laparoscopically; however, robotic-assisted surgery is increasingly utilized for revisional cases. Revisional procedures generally entail longer hospital stays compared to primary surgeries. Bariatric surgery is considered safe, with mortality rates below 1% reported in most national registries [24,25,26].

**Revisional Bariatric Surgery**

Revisional metabolic bariatric surgeries, as classified by the IFSO registry, refer to surgical interventions that modify or replace a previously performed bariatric procedure. These surgeries are recommended in cases of postoperative weight regain, inadequate weight loss, adverse side effects, or the recurrence of metabolic conditions. This highlights the chronic and relapsing characteristics of obesity [20,27,28]. Corrective surgeries address complications such as internal hernias or strictures [29,30,31]. Approximately 25,592 revisional procedures were reported globally, excluding 5435 cases in the United States. RYGB was the most common revisional procedure, valued for its efficacy in managing complications or suboptimal outcomes. Revision rates vary internationally, from 16.5% in France to as low as 2.6% in the United States, with an overall global revision rate of 6.5%. Excluding the U.S., this rate rises to 10%, possibly due to earlier reliance on procedures with higher revision rates like AGB and SG [20,32].

**Safety and Mortality Rates**

Perioperative mortality in bariatric surgery is low. Chang et al.’s systematic review found 30-day mortality rates between 0.08% and 0.22%, rising slightly postoperatively to 0.31–0.35%. Complication rates ranged from 10% to 17%, with reoperation rates of 6–7%; LAGB had the highest long-term reoperation rates [33]. Historical data (1995–2004) reported perioperative mortality at 0.9% and overall mortality at 2.6% [34]. In the UK, in-hospital mortality was 0.07%, and 30-day post-discharge mortality was 0.08%, making bariatric surgery safer than many other common surgical procedures such as knee or hip replacements [35]. According to the 8th IFSO registry, mortality for primary bariatric procedures ranged from 0.01% to 0.25%, compared to 0.05% to 1.42% for revisional surgeries [20].

**Guidelines on Indications for Metabolic and Bariatric Surgery**

Longitudinal studies provide strong evidence supporting the safety, effectiveness, and long-term benefits of metabolic and bariatric surgery (MBS) for managing severe obesity and its associated health conditions. These studies show a decrease in all-cause mortality when compared to non-surgical treatments. MBS is recommended for individuals with a body mass index (BMI) of 35 kg/m^2^ or higher, regardless of whether they have obesity-related health issues. It is also indicated for patients with a BMI of 30 kg/m^2^ or higher who have significant comorbidities, such as type 2 diabetes mellitus. Additionally, MBS may be considered for individuals with a BMI between 30.0 and 34.9 kg/m^2^ who have not experienced meaningful or sustained weight loss, or who have not seen enough improvement in their health conditions through non-surgical means. For older adults, eligibility for MBS should be assessed through a thorough evaluation of their comorbidity burden and frailty status [36,37]. Advances in perioperative care have facilitated the safe performance of MBS in progressively older populations, including septuagenarians [38]. While this cohort demonstrates a marginally increased incidence of postoperative complications relative to younger patients, substantial benefits in terms of excess weight reduction and remission of obesity-related diseases remain evident. Importantly, comorbidity profile, baseline obesity severity, and surgical modality are more predictive of both long-term outcomes and 30-day morbidity than chronological age alone [39].

Physiological changes associated with aging can impact the effectiveness of surgery, the rates of complications, and the recovery process after an operation. However, factors such as frailty, cognitive status, smoking habits, and overall functional capacity are more influential in determining postoperative risk than age alone. Notably, frailty has been linked to higher rates of negative surgical outcomes following metabolic and MBS. For elderly patients, it is essential to consider the potential benefits of MBS in relation to the risks of morbidity associated with untreated obesity [40,41]. In the pediatric and adolescent population, MBS should be considered for those with a BMI > 120% of the 95th percentile and severe comorbidities, or a BMI > 140% of the 95th percentile, following multidisciplinary assessment in a specialized bariatric center [42,43]. MBS serves as an effective supplementary intervention for patients with severe obesity who need major surgical procedures, such as joint replacement surgery, complex abdominal wall reconstruction, or organ transplantation. It is recommended to have a preoperative multidisciplinary consultation to optimize modifiable risk factors. This approach can help reduce perioperative complications and enhance overall surgical outcomes [44,45].

**Preoperative Evaluation**

Candidates eligible for bariatric surgery should undergo a thorough preoperative assessment, ideally managed by a multidisciplinary team that includes a bariatric surgeon, a registered dietitian, a behavioral medicine specialist, a cardiologist, a pulmonologist, an endocrinologist, and an anesthesiologist. This evaluation covers all aspects of surgical planning and perioperative care. It requires multiple consultations, educational interventions, structured planning, laboratory tests, diagnostic imaging, and targeted medical optimization based on the patient’s clinical condition [46].

Preoperative work-up routinely includes classification of physical status using the American Society of Anesthesiologists Physical Status (ASA PS) system. Patients with moderate obesity (BMI 30–40 kg/m^2^) are typically classified as ASA class II, whereas individuals with severe obesity (BMI ≥ 40 kg/m^2^) and significant comorbidities—such as type 2 diabetes mellitus, hypertension, or obstructive sleep apnea—are frequently categorized as ASA class III, depending on the extent and severity of systemic disease [47]. While ASA classification assists in surgical risk stratification and the formulation of appropriate perioperative strategies, it should not be employed in isolation; rather, it is best integrated into a comprehensive multidisciplinary risk assessment.

Higher ASA classification is linked to an increased risk of severe postoperative complications. However, preoperative functional capacity serves as a more reliable predictor of adverse outcomes and perioperative mortality. Therefore, using simpler and more practical assessments of functional status may provide greater clinical value in predicting perioperative risk for candidates undergoing bariatric surgery [48,49]. A complete medical history and thorough physical examination are essential, along with a detailed metabolic evaluation to identify and characterize obesity-related comorbidities. Documentation of unsuccessful attempts at medically supervised dietary interventions is usually required. Comprehensive preoperative profiling helps identify factors contributing to the patient’s obesity and informs individualized perioperative planning, which optimizes weight loss outcomes following surgery [50,51,52].

The preoperative evaluation is essential for confirming patient eligibility for bariatric surgery, ensuring that expectations are realistic, and assessing the patient’s commitment to the necessary behavioral and lifestyle modifications for achieving and maintaining long-term weight loss. This evaluation is clinically significant for several reasons. Obesity is often associated with comorbidities such as hypertension, obstructive sleep apnea, cardiovascular disease, and type 2 diabetes mellitus. A comprehensive preoperative assessment allows for the early identification and targeted management of these conditions, enabling the multidisciplinary team to tailor the surgical approach and perioperative care to each patient’s specific clinical profile [53,54,55]. Given that individuals with obesity face elevated perioperative risk compared to the general population, preoperative assessment is crucial for detecting preexisting medical conditions that could adversely influence anesthetic safety, surgical complexity, or postoperative recovery. Proactive enhancement of baseline health—including management of cardiometabolic disorders, enhancement of pulmonary function, and reduction in modifiable risk factors—decreases postoperative morbidity and mortality while increasing the probability of achieving and maintaining long-term weight loss outcomes [56,57,58].

## 2. Materials and Methods

### 2.1. Study Cohort

This study took place from June 2012 to June 2025 (14 years), all patients undergoing primary or revisional laparoscopic Roux-en-Y gastric bypass, gastric sleeve (SG), gastric balloon, gastric plication, or Single anastomosis duodeno-ileal bypass with sleeve gastrectomy (SADI-S) procedures were prospectively enrolled in a dedicated database [59,60]. Inclusion in the study required a minimum follow-up period of six months. Patients were stratified based on their surgical procedure for the analysis [61].

### 2.2. Eligibility and Preoperative Screening

We included patients aged 18 to 70 years who had previously attempted nonsurgical weight loss and who were considered medically, psychologically, and emotionally stable to undergo surgical and psychologically and emotionally stable treatment. We excluded patients who were pregnant or planning to become pregnant within 1 year of surgical treatment, who had a medical condition that would make surgery too risky (were psychologically unfit for surgery) [62,63,64]. Eligibility for bariatric surgery was verified according to the periodically updated American Society for Metabolic and Bariatric Surgery (ASMBS) and International Federation for the Surgery of Obesity and Metabolic Disorders (IFSO) guidelines (Table 1) [65].

Information collected for this study included demographics, comorbidities, type of bariatric surgery, concomitant procedures, and complications. All patients underwent routine preoperative screening conducted by a multidisciplinary team. Each patient was thoroughly assessed through comprehensive medical history, including substance use, alcohol and tobacco consumption, full physical examination. When suspected or inadequately treated conditions such as diabetes or dyslipidemia were identified, patients underwent appropriate investigations before surgical intervention. Specific preoperative evaluations included: obstructive sleep apnea screening polysomnography). Screening and management of comorbid conditions including hypertension, diabetes, and endocrine disorders. Esophago-gastro-duodenoscopy (EGD) to assess for Helicobacter pylori infection, laboratory tests, abdominal ultrasonography and a psychopathological evaluation through consultation with a psychotherapist [66,67,68]. For patients with complications, additional details were recorded, including time of diagnosis, diagnostic modality (computed tomography, endoscopy, ultrasound, biochemistry, etc.), length of hospitalization, treatment (conservative, endoscopic, or surgical), and follow-up.

### 2.3. Study Protocol

We applied standardized ASA and ERAS protocols both in the preoperative evaluation and in the management of patients enrolled in the study and who underwent bariatric surgery [69,70,71]. These protocols were also applied to patients who developed postoperative complications. In the evaluation of patients with complications and their management we applied Clavien-Dindo classification (Table 2) is a standardized system used to categorize surgical complications based on their severity and the required interventions [72].

For the patients enrolled in the study, the Enhanced Recovery After Surgery (ERAS) protocol was applied, which is an extensive, evidence-based, multimodal, and multidisciplinary protocol for surgery. ERAS aims to improve pre-intra- and postoperative physiology and optimize recovery after surgery. The concept of this approach has shown that a multidisciplinary tool that integrates several perioperative elements, with the use of minimally invasive surgery, can be adopted for various surgical procedures. Thus, the principles of these protocols and their advantages are extended to bariatric and metabolic surgery [73,74,75,76].

For this study, the sample size was determined by the total number of consecutive patients meeting the inclusion criteria and operated on during the study period, rather than by an a priori power calculation. Nevertheless, a post hoc power estimation was performed to assess the adequacy of the sample size for the primary outcome—postoperative complications within 30 days. Assuming an expected event rate of 10% and a clinically relevant absolute difference of 5%, a total of approximately 864 patients would have been required to achieve 80% statistical power (α = 0.05, two-sided). Thus, the inclusion of 1010 patients provided sufficient power to detect meaningful differences in postoperative outcomes.

### 2.4. Ethical Considerations

This study received approval from the local medical ethics committee of the ‘Sf. Spiridon’ Emergency Clinical Hospital, Iasi (approval number: 294/18 April 2023).

### 2.5. Statistical Methods

Data were collected and organized in Microsoft Excel (Microsoft Corporation, Redmond, WA, USA). Descriptive statistical methods were applied, including calculation of absolute and relative frequencies for categorical variables, and measures of central tendency (mean, median) for continuous variables. Results were summarized in tables and illustrated graphically using bar charts, and pie charts to facilitate the interpretation of trends and distributions. Qualitative data were analyzed at a 95% confidence level using the chi-square (χ^2^) test, a non-parametric method for comparing two or more frequency distributions. When applicable, odds ratios (ORs) were calculated to estimate the association between exposure and outcome variables.

## 3. Results

Between June 2012 and June 2025, 1010 patients underwent laparoscopic bariatric surgery in our center. 72% of patients were female, and the mean age was 39 years (range 18–70 years).

The most common obesity-related comorbidities among patients were hypertension, obstructive sleep apnea, dyslipidemia, hepatic steatosis, hepatomegaly, and diabetes mellitus.

**Figure 1:**
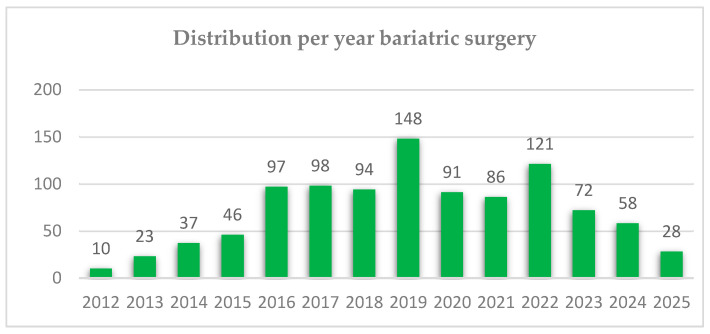
Distribution per year bariatric surgery.

The distribution of bariatric procedures on years is listed in Figure 1. The trend of bariatric surgery was increasing (y = 48.28 + 3.18x), predicting approximately 96 cases for the year 2026.

SG was the most common procedure (77.32%) followed by RYGB (17.52%), gastric plication (2.67%), SADI-S (1.98%), and gastric balloon (0.49%). For RYGB the alimentary limb length was 70 cm, and to create the biliopancreatic limb it was measured from the ligament of Treitz 150 cm.

**Figure 2:**
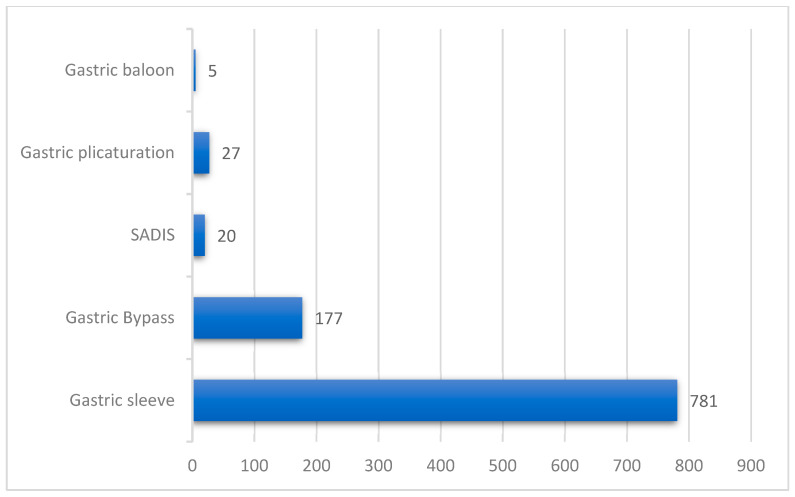
The type of surgical intervention.

Overall, the mean body mass index (BMI) was 42.29 kg/m^2^. A few cases of patients with BMI > 50 benefited from SADI-S bariatric surgery (Figure 2).

All procedures were successfully completed laparoscopically. All cases involved a 5-port approach and 60 mm linear staplers along with a 36-Fr were used. A standard leak test was performed, which involves instillation of 50 mL of methylene blue solution and passage of 50 mL of air through an orogastric tube. Additional reinforcement was applied to the long staple lines in the gastric sleeve and SADI-S procedure. Two drains were placed along the staple line. On postoperative day 2, all patients underwent a Gastrografin swallow to assess for a leak from the gastric staple line. If no problems were identified, patients were discharged home with dietary instructions.

The duration of the surgical intervention varied depending on the type of surgery, ranging from 45 min for gastric sleeve, 70 min Gastric Bypass, and 150 min for SADIS. The average length of hospital stays for patients who underwent bariatric surgery was 4 days, and for patients who experienced postoperative complications, the average length of hospital stay was 7 days. There was no postoperative mortality. The type of procedures from each year are described in Table 3.

**Complications analysis**

Patients were prospectively assessed for early (<30 days) and late (>30 days) postoperative complications through clinical examination, structured anamnesis, and routine investigations. For any medical/surgical emergency, patients came for evaluation or were directed to the hospital where the bariatric surgery was performed. Early complications were documented during hospitalization and at scheduled follow-up visits (7, 14, and 30 days postoperatively). Data collection included both surgical and medical events such as postoperative hemorrhage, kinking, trocar hernia, acute kidney injury.

There are several known risk factors for early postoperative complications: age between 30 and 40 years, gender (more frequent in men), BMI ≥ 40 kg/m^2^ or ASA class ≥ III. Comorbidities such as diabetes, obstructive sleep apnea, gastroesophageal reflux disease, hyperlipidemia, hypertension, prior abdominal surgery, anticoagulant therapy, oral analgesic use, and recent smoking history were also identified as risk factors [77]. Late complications (>30 days) were evaluated at follow-up visits at 3, 6, and 12 months postoperatively.

A structured assessment was conducted to identify delayed surgical or metabolic complications, including perforated ulcer, internal hernias, and nutritional deficiencies. This evaluation was complemented by laboratory investigations (complete blood count, electrolytes, liver function, and micronutrient levels) and imaging or endoscopic studies, when clinically indicated. Each reported event was independently reviewed by the bariatric surgical team and documented in the institutional electronic database.

This structured postoperative assessment based on clinical evaluation and anamnesis ensured standardized and reproducible reporting in accordance with ASMBS and IFSO recommendations [65].

**Figure 3:**
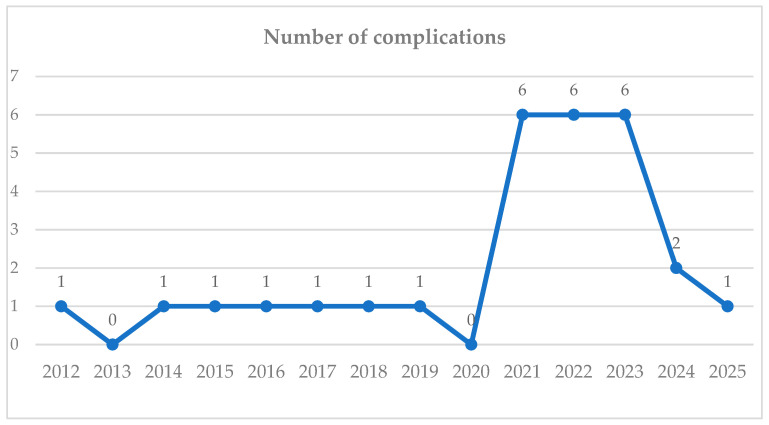
Number of complications per year.

Between 2012 and 2025, the number of acute complications per year was about 0 to 6, with an increasing trend (y = 0.04 + 0.27x), predicting approximately 4 cases for 2026 (Figure 3).

The observed increase in cases during the 2021–2023 interval parallels the expansion of Roux-en-Y gastric bypass (RYGB) surgery. Within this context, we identified a higher incidence of simultaneous hemorrhage from both the gastrojejunal and jejunojejunal anastomoses. These events occurred predominantly in patients with significant comorbidity burden and lifestyle-related risk factors, including cardiovascular and hematologic disorders, smoking, and NSAID consumption.

The complications represent 6.73% of total cases, 2.77% in male and 3.96% in female (*p* = 0.015). By gender, the odds ratio (OR) of the complications was significantly higher in male than in female (9.79% vs. 5.52%; OR = 1.89; IC95%:1.14–3.12; *p* = 0.015).

The number of early complications was significantly high in male than in female (6.71% vs. 1.24%; OR = 5.74; IC95%:2.57–12.85; *p* = 0.001). The number of late complications was not significant between gender (female 4.40% vs. male 3.18%; *p* = 0.337) (Table 4).

Among intraluminal hemorrhages, the gastrojejunal anastomosis represented the most frequent bleeding site (77.77%), followed by the jejunojejunal anastomosis. Extraluminal bleeding was primarily associated with internal hemorrhage (86.66%), while port-site bleeding was identified in 2 patients (13.34%). Overall, intraluminal sources accounted for fewer cases compared to extraluminal bleeding, suggesting that most postoperative hemorrhagic events originated from intra-abdominal sources rather than anastomotic leakage (Table 5).

Postoperative bleeding cases are distributed across age groups and gender, with most occurrences in patients aged 31–60 years and a slight predominance among females (Table 6).

Table 7 summarizes the thirty-day postoperative complications stratified according to the Clavien–Dindo classification system. Most adverse events were grade IIIb complications, requiring surgical or endoscopic intervention under general anesthesia, predominantly represented by intraluminal and extraluminal bleeding (0.89% and 1.48%, respectively). Minor complications such as acute kidney injury (grade I) and isolated cases of kinking or trocar-related hernia were rare. Overall, the incidence of postoperative morbidity within 30 days remained low (2.57%), indicating a favorable short-term surgical outcome.

## 4. Discussion

**Bleeding After Bariatric Surgery**

Patients who presented complications after bariatric surgery were investigated by imaging (ultrasound, computed tomography), laboratory tests, upper digestive endoscopy.

Major postoperative bleeding is defined as a decrease in hemoglobin > 2 g/dL or clinically revealed bleeding externalized on drain tubes or for cases of intraluminal digestive bleeding externalized by hematemesis, melena, hematochezia, requiring intervention (blood transfusion, endoscopic intervention or surgery). According to Heneghan et al. postoperative bleeding is the most documented complication, occurring in 0.4–4.4% of cases following a gastric bypass and 0.4–3.4% following a vertical gastrectomy [78].

Clinically, the patient with signs of postoperative bleeding becomes hemodynamically unstable, hypotensive and tachycardic with cold sweats.

In cases of intraluminal or extraluminal hemorrhage, low-molecular-weight heparin was discontinued, and patients were closely monitored hemodynamically, with correction of hydroelectrolytic and acid-base imbalances. For intraluminal hemorrhage located at the gastro-jejunal and jejuno-jejunal anastomosis (endoscopic instruments facilitated precise unlooping and retraction maneuvers, permitting progression to the jejuno-jejunal anastomosis), upper digestive endoscopy and endoscopic hemostasis were performed using sclerotherapy—adrenaline, bipolar coagulation, mechanical hemostasis—clips with obtaining hemostasis.

For patients who had extraluminal hemorrhage, laparoscopic surgery was performed again and hemoperitoneum evacuation and drainage were performed, without identifying the source of active bleeding at the time of reoperation.

Patients with trocar site hemorrhage underwent hemostasis using a fascia closure device and a slowly absorbable suture (Vicryl, synthetic absorbable thread).

**Kinking**

Kinking is small bowel obstruction at or near the jejuno-jejunal anastomosis is a rare complication after gastric bypass, occurring in less than 1% of patients (0.84%) according to Annick E. Taselaar et al. [79]. Obstruction can occur either due to abnormal folding (kinking) of the anastomosis or due to narrowing of the anastomosis at the site of the enterotomy closure due to technical error. Kinking is likely associated with the closure of the e jejunojejunostomy defect, as its anatomical proximity to the anastomosis increases the risk of distortion or tension. Because the distal anastomosis includes both the alimentary and biliopancreatic branches of the bypass, obstruction at this location can present with very different symptoms.

Obstruction of the alimentary branch will result in nausea, vomiting, and absence of intestinal transit for gas, while obstruction of the biliopancreatic branch will result in dilatation of the gastric remnant [80]. Diagnosis was made by CT scan. Treatment was laparoscopic surgery of the obstruction with jejuno-jejunal anastomosis repair.

**Incisional Hernia**

Trocar site hernia (incisional hernia) appears to be a rare complication of laparoscopic surgery. Regarding the incidence of incisional hernia after bariatric surgery, most authors report an incidence between 0.2 and 1%. Schauer et al. presented two cases (0.7%) [81] and Chevallier et al. demonstrated that 4 (0.4%) out of 1000 patients operated had incisional hernia [82,83].

Trocar hernias were repaired laparoscopically using fascia closure device (Vicryl suture, synthetic, absorbable suture).

**Perforated Ulcers**

Perforated ulcers after sleeve gastrectomy are rare. Cases of ulcer perforation in the gastric remnant and the excluded segment of the duodenum after RYGB are cited in the literature, being rare. Only 29 other cases are reported in the literature, with a reported prevalence of perforation between 0.12% and 0.84% [84]. Among these cases, 21 (72%) patients had perforation of a duodenal ulcer, 7 (24%) had perforation of a gastric ulcer, and 1 (3.4%) had simultaneous perforation of both a duodenal and gastric ulcer [85].

**Dumping syndrome**

Dumping syndrome, a frequently described phenomenon after bariatric surgery, arises from the rapid emptying or “dumping” of undigested gastric contents into the small intestines [86]. According to Cawley et al. in general, this complication occurs on average in 14.6% of patients after bariatric surgery [87]. Symptoms appeared in patients on average after two years of RYGB [86].

**Gastro-Esophageal Reflux Disease**

Some studies suggest that 20–30% of patients complain of GERD after RYGB [88,89] and several hypotheses have been formulated to explain GERD symptoms after RYGB. Firstly, the persistence of acid-secreting parietal cells in the gastric pouch [90]. Secondly, the potential endoscopic evidence of bile reflux in the pouch in some patients complaining of upper gastrointestinal symptoms [91]. Thirdly, impaired motility of the Roux limb as hypothesized by Rebecchi et al. who demonstrated a high rate of esophagitis after RYGB, a high number of weakly acidic reflux at pH-impedancemetry, with no abnormalities in lower esophageal sphincter pressure or body motility [92]. Fourthly, the appearance of a hiatal hernia with pouch migration in the mediastinum [93]. After RYGB, even duodenogastric bile reflux to the excluded stomach has been demonstrated in 36% of patients, exposing the gastric mucosa in the excluded stomach to the potential deleterious effects of bile, with uncertain clinical significance [89,94].

The anatomical and physiological mechanisms underlying the development or exacerbation of gastroesophageal reflux disease (GERD) following sleeve gastrectomy (SG) are multifactorial. In cases of de novo GERD, proposed contributing factors include delayed gastric emptying, reduction in lower esophageal sphincter (LES) pressure, attenuation of the angle of His, decreased gastric compliance and reservoir capacity, and elevated intragastric pressure resulting from the creation of a narrow tubular gastric pouch. Additionally, partial herniation of the gastric sleeve into the mediastinum may further predispose to reflux [95].

**Gallstones**

According to Chen et al. gallstones represent a common yet often underappreciated complication following bariatric surgery, with reported incidence rates ranging widely from 10.4% to 52.8% within the first postoperative year. Multiple factors contribute to gallstone formation in this setting, including intraoperative injury to the hepatic branch of the vagus nerve, alterations in bile composition, reduced food intake, shifts in gastrointestinal hormone levels, and dysbiosis of the gut microbiota [96].

Patients had treatment recommended—pharmacological interventions such as ursodeoxycholic acid (UDCA), which has demonstrated efficacy in reducing gallstone incidence and the need for subsequent cholecystectomy [97].

**Internal hernia**

Roux-en-Y gastric bypass (RYGB), representing the sole occurrence of this complication within the study cohort. The diagnosis was made by CT scan imaging and segmental enterectomy and laparoscopic anastomosis were performed.

Petersen’s space hernias are the most common location for internal hernias following laparoscopic gastric bypass surgery. Petersen’s hernia occurs when intestinal loops protrude through the space created between the mesentery of the alimentary limb and the transverse mesocolon, according to El Nogoomi et al. with an estimated incidence that ranges from 0.9% to 4.5% [98].

**Acute kidney injury**

Acute kidney injury (AKI) usually appears a month after bariatric surgery and is characterized by high creatinine levels in relation to the patient’s weight. The incidence of early postoperative AKI after bariatric surgery is about 1% [99].

**Nutritional Deficiency**

Iron deficiency, expressed by low serum ferritin, occurs in more than 30% of patients after 5 years from surgery, with a similar rate after RYGB and SG, as recently reported by Alexandrou et al. [100].

As described in the study by Nor Hanipah et al. Vitamin B12 deficiency is a major cause of anemia in patients who undergo RYGB, with a prevalence of 19–35% after 5 years [101]. The prevalence of folic acid deficit after both restrictive and malabsorptive procedures ranges from 9% to 39% as reported by von Drygalski et al. [102]. As described in the study by Lupoli et al. the incidence of calcium deficiency after surgery is almost 10% and is caused by reduced calcium absorption that results from bypassing the duodenum and proximal jejunum, which are the main sites of absorption. The prevalence of hypovitaminosis D after surgery varies between 25% and 73%. Protein malnutrition remains the most severe macronutrient complication associated with malabsorptive surgical procedures. It has been reported in 7–21% of patients [103].

**Gastric Balloon**

Minimally invasive obesity treatment with gastric balloon placement carries inherent risks, with a notable incidence of adverse events reported in the literature. Compared with laparoscopic bariatric surgery, balloon therapy has been associated with higher overall complication rates and a mortality rate reported to reach 0.05% in some series. Early complications may include gastrointestinal bleeding, esophageal tears or perforation, and pneumonia. Late complications can involve gastric outlet obstruction following balloon deflation, acute pancreatitis, gastric ischemia, ulceration, and, in severe cases, perforation [104]. The overall complication rate reported by Fittipaldi-Fernandez et al. is 7.32% [105]. No patients required premature balloon removal due to intolerance in the present study and no complications were observed among patients who underwent gastric balloon placement.

**Without fistula**

Despite the rising global volume of bariatric and metabolic surgeries, overall mortality and adverse event rates including anastomotic leaks and fistulas have declined over the past two decades. This improvement is largely attributable to advancements in surgical techniques such as laparoscopy and robotic-assisted procedures, the use of enhanced surgical materials, and increased surgeon experience [106].

The incidence of leaks and fistulas is comparable across the most frequently performed bariatric procedures, ranging from 0.4% to 5.6% for RYGB and 1.9% to 5.3% for SG, with higher rates observed following revisional surgery. In high-volume, specialized centers, reported leak rates can be as low as 0.5% [107,108].

In the cohort analyzed from our bariatric surgery center, no cases of postoperative fistula formation were identified, and no mortality events were recorded throughout the study period. These results highlight the favorable safety profile achievable specialized bariatric units, where procedures are performed by experienced surgical teams and supported by comprehensive multidisciplinary care. Rigorous adherence to standardized preoperative assessment, meticulous operative technique, and structured postoperative monitoring likely contributed to these outcomes. The absence of such severe complications reinforces the importance of specialized expertise and optimized perioperative protocols in minimizing risk and improving patient safety in bariatric surgery practice.

**Study Limitations, Challenges and Strengths**

This study presents several inherent challenges. The single-center setting and potential variations in surgical technique and patient comorbidities could affect the generalizability of the findings. Despite these limitations, the study has notable strengths. The relatively large cohort of 1010 patients provides a comprehensive overview of postoperative complications following bariatric surgery. The study offers valuable insights into real-world outcomes in a specialized bariatric surgery setting.

Nevertheless, the application of the Clavien–Dindo classification system, and a detailed evaluation of complication patterns in relation to risk factors represent significant methodological strengths.

## 5. Conclusions

The clinical outcomes of bariatric surgery are strongly influenced by the systematic identification and optimization of modifiable risk factors, adherence to standardized preoperative protocols, and rigorous postoperative monitoring. Moreover, surgical expertise and the operative technique play a pivotal role in minimizing complications. Enhancing perioperative risk prediction and improving patient outcomes requires a multidisciplinary, protocol-driven approach across all phases of care.

## Figures and Tables

**Table 1:** Inclusion and Exclusion Criteria for Bariatric Surgery. Adapted from Eisenberg D. et al. [65].

Inclusion Criteria	Exclusion Criteria
**Body mass index (BMI) ≥ 40 kg/m^2^, irrespective of obesity-related comorbidities**	Severe, uncontrolled psychiatric disorders (e.g., active psychosis, untreated major depression, active eating disorders such as bulimia nervosa or uncontrolled binge eating)
**BMI ≥ 35 kg/m^2^ with at least one major obesity-related comorbidity (type 2 diabetes mellitus, uncontrolled hypertension, moderate-to-severe obstructive sleep apnea, severe dyslipidemia, non-alcoholic steatohepatitis with advanced fibrosis, obesity-related cardiovascular disease)**	Severe uncontrolled medical conditions contraindicating anesthesia or surgery (e.g., advanced heart failure, end-stage pulmonary disease)
**BMI ≥ 30 kg/m^2^ with poorly controlled type 2 diabetes mellitus or severe metabolic risk factors**	Active alcohol or substance use disorder
**Documented failure of conservative therapy (diet, lifestyle modification, pharmacotherapy, behavioral therapy)**	Active malignancy requiring current oncologic treatment
**Age 18–70 years (with exceptions for selected adolescents and older adults after multidisciplinary evaluation)**	Severe, uncorrectable coagulopathy
**Demonstrated motivation and capacity to comply with long-term postoperative follow-up**	Lack of adherence to medical care or inability to understand the implications of surgery
**Willingness to adopt permanent lifestyle modifications and lifelong nutritional supplementation**	Technical or anatomical contraindications (rare, e.g., severe gastrointestinal pathology precluding reconstruction)

**Table 2:** Clavien-Dindo classification. Adapted from Bolliger et al. [72].

Grade	Description
I	Any deviation from the normal postoperative course without the need for pharmacological treatment or surgical, endoscopic and radiologic interventions.
	Acceptable therapeutic regimens are drugs, such as antiemetics, antipyretics, analgesics, diuretics and electrolytes, and physiotherapy.
	This grade also includes wound infections opened at the bedside.
II	Requires pharmacological treatment with drugs other than those allowed for grade I complications. Blood transfusions, antibiotics and total parenteral nutrition are also included.
III	Requires surgical, endoscopic or radiological intervention.
IIIa	Intervention under regional/local anesthesia.
IIIb	Intervention under general anesthesia.
IV	Life-threatening complication requiring intensive care/intensive care unit management.
IVa	Single-organ dysfunction.
IVb	Multiorgan dysfunction.
V	Patient demise.

**Table 3:** Type of bariatric procedures per year.

Year	Number	Type Procedures
2012	10 (0.99%)	SG 9 Gastric plications 1
2013	23 (2.27%)	SG 20 Gastric plications 3
2014	37 (3.66%)	SG 36 Gastric plications 1
2015	46 (4.55%)	SG 45 Gastric ballon 1
2016	97 (9.60%)	SG 92 RYGB 5
2017	98 (9.70%)	SG 83 RYGB 10 Gastric plications 5
2018	94 (9.30%)	SG 90 RYGB 3 Gastric plications 1
2019	148 (14.65%)	SG 120 RYGB 13 Gastric plications 11 SADI-S 2 Gastric ballon 2
2020	91 (9%)	SG 63 RYGB 15 SADI-S 7 Gastric plications 4 Gastric ballon 2
2021	86 (8.51%)	SG 36 RYGB 38 SADI-S 11 Gastric plications 1
2022	121 (11.98%)	SG 77 RYGB 44
2023	72 (7.12%)	SG 48 RYGB 24
2024	59 (5.84%)	SG 44 RYGB 15
2025	28 (2.77%)	SG 18 RYGB 10

**Table 4:** Postoperative bariatric complication.

Early Complication (<30 Days)	Number of Cases	Late Complication (>30 Days)	Number of Cases
**Intraluminal bleeding**	9 (3F, 6M)	Perforated ulcer	1M
		Dumping	7 (5F, 2M)
		GERD	8F
**Extraluminal bleeding**	15 (4F, 11M)	Gallstones	7F, 2M
**Kinking**	1F	Internal hernia	1F
**Trocar–related hernia**	1F	Trocar–related hernia	2 (1F, 1M)
**Acute kidney injury**	2M	Nutrient deficiency	12 (9F, 3M)
**Total (%)**	28 (2.77)	Total (%)	40 (3.96)
**Total %**	2.77%	Total %	3.96%

F—Female, M—Male.

**Table 5:** Bleeding after bariatric surgery.

Bleeding	Source	Number of Cases
Intraluminal	Gastrojejunal anastomosis	7
	Jejuno-jejunal anastomosis	2
Extraluminal	Port site bleeding	2
	Internal bleeding	13

**Table 6:** Association of post-operative bleeding with age groups and gender.

Age Groups (Years)	18	18–30	31–40	41–50	51–60	61–70	>70
Gender	0	3F, 1M	4F, 4M	3F, 2M	2F, 3M	2M	0

F—Female, M—Male.

**Table 7:** Thirty-Days Complication grade by the Clavien-Dinco Classification.

Complications	I	II	IIIa	IIIb	IV	V	Total	%
Intraluminal bleeding	0	0	0	9	0	0	9	0.89
Extraluminal bleeding	0	0	0	15	0	0	15	1.48
Kinking	0	0	0	1	0	0	1	0.09
Trocar–related hernia	0	0	0	1	0	0	1	0.09
Acute kidney injury	2	0	0	0	0	0	2	0.19
Total	2	0	0	26	0	0	28	0
%	0.19	0	0	2.57	0	0	0	0

## Data Availability

Researchers interested in accessing anonymized data may submit a request to the corresponding author, in accordance with Ethics Committee approval.
